# Gut Microbiota: Target for Modulation of Gut-Liver-Adipose Tissue Axis in Ethanol-Induced Liver Disease

**DOI:** 10.1155/2022/4230599

**Published:** 2022-05-20

**Authors:** Dhara Patel, Dixa Sharma, Palash Mandal

**Affiliations:** PD Patel Institute of Science and Technology, Charotar University of Science and Technology, 388421, Changa, Gujarat, India

## Abstract

Consumption of alcohol (ethanol) in various forms has been an integral part of human civilization. Since ages, it also has been an important cause of death and health impairment across the globe. Ethanol-mediated liver injury, known as alcoholic liver disease (ALD), is caused by surplus intake of alcohol. Several studies have proposed the different pathways that may be lead to ALD. One of the factors that may affect the cytochrome P450 (CYP2E1) metabolic pathway is gut dysbiosis. The gut microbiota produces various compounds that play an important role in regulating healthy functions of distal organs such as the adipose tissue and liver. Dysbiosis causes bacteremia, hepatic encephalopathy, and increased intestinal permeability. Recent clinical studies have found better understanding of the gut and liver axis. Another factor that may affect the ALD pathway is dysfunction of adipose tissue metabolism. Moreover, dysfunction of adipose tissue leads to ectopic fat deposition within the liver and disturbs lipid metabolism by increasing lipolysis/decreasing lipogenesis and impaired glucose tolerance of adipose tissue which leads to ectopic fat deposition within the liver. Adipokine secretion of resistin, leptin, and adiponectin is adversely modified upon prolonged alcohol consumption. In the combination of these two factors, a proinflammatory state is developed within the patient leading to the progression of ALD. Thus, the therapeutic approach for treatments and prevention for liver cirrhosis patients must be focused on the gut-liver-adipose tissue network modification with the use of probiotics, synbiotics, and prebiotics. This review is aimed at the effect of ethanol on gut and adipose tissue in both rodent and human alcoholic models.

## 1. Introduction

In 2018, according to World Health Organization (WHO) global report on alcohol and health, nearly 3 million people died in the year 2016 due to abuse of alcohol [1]. Alcohol consumption has been found to be one of the major causes of organ dysfunction and tissue injury leading to the ALD, cancer, compromised immune system, pancreatitis, heart diseases, and disturbance in circadian clock [2]. Although alcohol is primarily necessary for organ dysfunction, it is not the only factor causing ALD. Subsequently, other factors that contribute to the toxicity of alcohol pathology are the alcohol-induced modifications in the composition and function of the gastrointestinal tract (GIT) microbiota and function of adipose tissue.

The GIT contains trillions of microbes with more than 1000 different species; the major groups from the vast phyla are *Firmicutes* and *Bacteroidetes* [3]. There is a symbiotic relationship between the microbes of the intestine and GIT. Gut microbiota helps in the extraction of energy and synthesis of amino acids and vitamins from food as well as maintaining the vital barriers against pathogens and therefore maintaining the general homeostasis of a healthy human being. With disturbance in the healthy microbiome of GIT, the homeostasis might also be affected and prolonged alteration may cause ALD, inflammatory bowel disease (IBD), type I and II diabetes, and cardiovascular disease [2]. However, it is not yet evident whether dysbiosis is the cause of the disease or the outcome of disease.

Chronic alcohol consumption leads to lipolysis and ectopic fat deposition in the liver which signifies the importance of function of adipose tissue in progression of ALD [3]. Function of adipose tissue derived fatty acids and lipid metabolism is impaired due to chronic alcoholism. Moreover, in long-term exposure to alcohol, lipogenesis is decreased and lipolysis is increased leading to fat loss [4, 5]. White adipose tissue (WAT) is an endocrine organ which releases several adipokines such as adiponectin, leptin, and resistin which are modulated negatively in ALD [6, 7]. Tumor necrosis factor alpha (TNF-*α*), Interleukin-6 (IL-6), and Monocyte chemoattractant protein-1 (MCP-1) are essential modulators of lipid metabolism and are affected by chronic alcoholism [8]. Lastly, a proinflammatory state developed within WAT contributes to lipodystrophy resulting in fat deposition in the peripheral organs, which enhances the pathological state of ALD. Therefore, this review focuses on the current knowledge of regulation of gut microbiota composition and adipose tissue in ALD.

## 2. Gut Microbiota Composition Analyses with Intake of Alcohol

Due to the vast genetic diversity, it is incredibly difficult for absolute characterization of microbial communities in the GIT. Thus, researchers use molecular tools such as RNA, DNA, and PCR-based target approaches from colonic tissue biopsies and fecal samples to analyze these complex GIT communities. A supporting study of four healthy individuals on microbiota community in the stomach, mouth, duodenum, colon, and stool showed that the context of GIT and feces is mostly similar. However, three of four individuals had reduced number of *Bacteroidetes* in fecal samples, resulting in the alteration of the *Firmicutes* and *Bacteroidetes* ratio, which is used as a diagnostic parameter for ALD [9].

An inadequate study has been performed on correlating the structure and activity of GIT microbiota under the influence of alcohol as compared to other effects on the body. Previous studies suggest that C57BL/6 mice that were fed 30.9 g/kg per day of alcohol for 3 weeks have developed ALD as compared to the control group. The study observed the bacterial overgrowth in a small intestine and cecum dysbiosis [10]. In another rat model study, 10-week alcohol feeding (8 g/kg/day) experiment found the occurrence in dysbiosis with alcohol induced endotoxemia [11]. These studies evidently suggested that alteration of microbiota, intestinal permeability, proinflammatory factors, and endotoxemia may potentially contribute to liver pathology or intestinal dysbiosis [12].

Supporting evidences were also found in humans. A study of shotgun metagenomic sequencing elucidated that the sensitive microbial pathways are altered consistently with the degree of hepatic steatosis in the patients who have stop drinking alcohol for 2 weeks [13]. A study reported that minimal hepatic encephalopathy (MHE) and liver cirrhosis have found overgrowth of aerobic and anaerobic bacteria in the small intestine using a culture-based method [14]. Another study of sigmoid biopsies from alcoholic and healthy individuals showed alteration of mucosa associated microbiota [15]. An *in vivo* study also suggests the detection of higher level of endotoxin and bacterial products in the blood circulation, signifying the hyperpermeability of the intestinal lumen [16]. Evidential increase in the families of *Prevotellaceae*, *Enterobacteriaceae*, *Veillonellaceae*, and *Streptococcaceae* was observed in the alcoholic cirrhosis patients as compared to the hepatitis B control group [17]. Another interesting study suggests the microbial community difference in the alcoholics and alcoholic cirrhotic patients. Apparently, alcoholic without cirrhosis patients have shown decrease in *Veillonellaceae* and *Clostridia* spp. while alcoholic with cirrhosis patients have shown an increase in *Veillonellaceae*, *Prevotellaceae*, *Enterobacteriaceae*, and *Fusobacteria* which may affect the prognosis of the patient condition [18]. These differences in finding of bacterial community between alcoholics and alcoholic cirrhosis may result in the progression of the liver disease or may act as a biomarker for the same. More studies are required to determine the relationship between the microbiota and liver diseases under the influence of chronic alcohol.

Alcohol consumed in certain amount can be beneficial for GIT microbial composition. One such study was performed where red wine (272 mL per day), dealcoholized red wine (272 mL per day), or gin (100 mL per day) for 20 days was consumed by the patients. Red wine and dealcoholized red wine consumption increased the abundance of beneficial bacteria *Bifidobacterium* in the GIT. Consumption of gin increases *Clostridium* when compared to dealcoholized red wine drinking which increases *Fusobacteria* [19]. It has been evident that consumption of polyphenols is linked with growth in bacteria which are known to promote healthy ecosystem of GIT. Thus, they can be utilized as dietary supplements to alter the bacterial community in a specific way. Additionally, regular intake of red wine polyphenols rises the growth of *Bifidobacterium* which could be allied as a prebiotic effect on gut microbiota [20]. A study on alcoholic hepatitis patients has shown negative correlation between liver disease score and Shannon diversity; further relative abundance of *Akkermansia* is decreased, and that of *Veillonella* is increased. Also, antibiotic-treated patients have shown reduction in *Bacteroides* and Shannon diversity, while patients on steroids have increase in *Veillonella* abundance. It was signified that the modification in the gut microbiome in alcoholic hepatitis patient is distinct and can be an attractive target for prevention or treat ALD [21]. Alcohol altering the gut microbiota or the disturbance in gut microbiota leading to progression of ALD is yet to be completely understood and recognized.

## 3. Overview of the Dysbiosis Linkage in ALD Progression

The pathological onsets during chronic consumption of alcohol are gradual, with the change in the gut microbiota under the persistent influence of alcohol [20]. Studies have shown that the oxidative stress in the intestinal lumen caused by alcohol consumption disrupts the tight junction of the intestine leading to intestinal hyperpermeability [20], due to which the translocation of gram-negative bacteria and its products occur through portal vein circulation. Exposure to such endotoxins can cause inflammation in the liver, which would add to the conjunction effect of direct alcohol and cause ALD. Overgrowth of bacteria or translocation of the bacterial products or metabolites may cause infection and may result in mortality of the ALD cirrhotic patients [22]. It has also been reported that leaky gut alone did not explain increased microbial translocation in patients. Rather, duodenal dysbiosis with an increase in *Streptococcus*, *Shuttleworthia*, and *Rothia* leads to intestinal permeability and elevated markers of microbial translocation in alcoholic disorder patients with progressive ALD [23]. A study on peroxisome proliferator-activated receptors-delta (PPAR *δ*) suggests that activation of PPAR *δ* agonist seladelpar elevates proliferation of epithelial cells in the small intestine and suppresses macrophage-derived inflammation as a result of stabilizing gut barrier function and dysbiosis [24]. Gut signifies a vital role in the prognosis of ALD and therapeutic approaches.

## 4. *Lactobacillus* Used to Modify Gut Microbiota in Alcoholic Liver Disease

Probiotic and synbiotic intervention may modify the dysbiosis caused by ALD. Probiotics are defined as live organisms that are beneficial to host more than their nutrition value [25], while synbiotics are combinations of prebiotic (nondigestible fibers) and probiotic that stimulate growth of microbiota in the intestine. *Lactobacillus rhamnosus* GG (LGG) is one of the most studied probiotic bacterial strains which is known to be effective for intestinal development and immunity, ameliorate diarrhea, ulceration, colitis, and improved intestinal barrier function [26, 27].

A study suggested that with the administration of LGG (2.5 × 10^7^ cfu/mL), oats (10 g/kg) along with alcohol (8 gm/kg/day) for 10 weeks in SD rats resulted in the prevention of alcohol associated dysbiosis [9]. Amelioration of intestinal hyperpermeability and oxidative stress are few of the factors that altered the progression of alcohol steatohepatitis [28]. Another study also supports the beneficial role of LGG, where 1 mL of LGG along with *Lieber-DeCarli* Diet, with and without 5% (*w*/*v*) alcohol, was found to decrease the count of *Bacteroidetes* but *Firmicutes*, *Proteobacteria*, and *Actinobacteria* increased considerably [29]. However, LGG administration did increase the *Firmicutes* along with *Lactobacillus*, while other studies have suggested the prevention of ALD by maintaining the gut permeability, endotoxemia, and liver injury [30, 31]. Human studies with LGG administration show that minimal hepatic encephalopathy (MHE) patients with cirrhosis do have beneficial microbial growth but do not have increased *Lactobacillus* or improved cognitive function [14]. The above-mentioned studies can confirm that use of probiotic or synbiotics can alter the host microbiota in a more beneficial way for the clinical trial. With the gut dysbiosis, the second hit of alcohol consumption is on the adipose tissue in ALD.

## 5. Adipose Tissue Metabolism in the Presence of Alcohol

**Figure 1:**
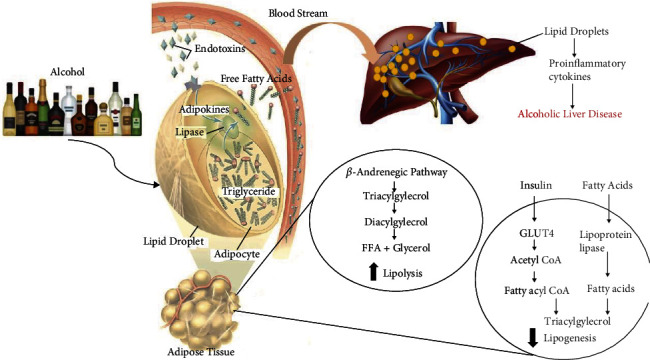
Effect of ethanol on adipose tissue contributing to the advancement of alcoholic liver disease condition.

The progression of ALD is a multifactorial disease condition. The above-mentioned facts indicate that prevention is better than cure as the life expectancy of the patients after clinical diagnosis of alcoholic steatohepatitis (ASH) is very short [32, 33]. The known deleterious consequences of alcohol from hepatic oxidative stress, inflammation, and cell apoptosis may not be the only route for the progression of ALD. Adipose tissue is mainly considered the primary organ for storage, but the recent advancement has discovered that adipokines have led to considering the white adipose tissue (WAT) as a major endocrine organ [34–36]. Thus, dysfunction of adipose tissue might be correlated with the pathophysiology of many metabolic diseases including alcoholic liver disease as shown in Figure 1 [37].

Dysfunction of adipose tissue may affect the hepatic metabolism by adipocyte cell death and inflammation release of free fatty acids (FFA) [8]. Simultaneously, release of endotoxin from the compromised gut microbiota in portal circulation may play an important role in mediating inflammatory responses and liver injury in the presence of alcohol [38–42]. Thus, the dual hit of inflammation in adipose tissue and leaky gut plays a vital role in the progression of ALD.

The process of production of FFA and glycerol from the hydrolysis of triglyceride as an energy source by other tissues during inflammation is known as lipolysis [43]. These circulating FFA are normally removed by the liver. An *in vivo* radio-labeled triglyceride study suggested the increase of lipolysis in adipose tissue upon intake of chronic ethanol [37, 44]. Catecholamine like epinephrine and norepinephrine stimulation via B adrenergic receptor is a potential activator for lipolysis upon intake of alcohol [45]. Lipolysis is also activated by fibroblast growth factor 21 (FGF21) to reduce accumulation of lipid via peroxisome proliferator-activated receptor gamma (PPAR*γ*) and CCAT-enhancer-binding protein (C/EBP). WAT is mainly responsible for the secretion of FGF21 as energy responsive adipokines in the presence of glucose metabolism in the adipose tissue [44].

To support the role of FGF21, a deficiency of FGF21 upon intake of chronic-binge alcohol resulted in the increase of plasma and eWAT FGF21 expression, and increase in lipolysis was prevented [45]. A contradicting study in FGF21 knockout mice indicated the decrease in plasma catecholamine concentration and eWAT mass [43]. The role of FGF21 as a metabolic regulator upon alcohol intoxication requires further investigation. In contrast to lipolysis, the process of energy storage is known as lipogenesis, while effects of alcohol on lipolytic surpass its lipogenic effects.

Alcohol does alter the lipogenic pathway. After chronic ethanol consumption, PPAR*γ* decreased in WAT, which is known as a prominent lipogenic stimulant [46–48]. The mitogen-activated protein kinase (MAPK) pathway regulates PPAR*γ*. Thus, the partial suppression of MAPK helps to restore levels of PPAR*γ* [49]. The effect of chronic alcohol intake on modulators of lipid metabolism relies on the *in vivo vs. in vitro* model system. Finally, *in vivo* administration of labeled triglyceride into chronic ethanol fed rats did not indicate any significant difference in the synthesis of triglyceride [37], since early changes developed by alcohol intake give insight into the initiation of long-term effects induced by lipodystrophy.

Adipose tissue also contains visceral adipose tissue (VAT) which is also affected upon intake of alcohol. In one of the studies, the possible mechanism upon alcohol induction suggested that increase in VAT and hypertrophic adipocytes leads to hypoxia which induces factor-1A (HIF-1A) and GLUT1 activation resulting in inflammation of the adipocytes and secretion of inflammatory adipokines such as leptin, TNF-*α*, and IL-6, which are vital for the prognosis of ALD [50, 51].

Binge or chronic alcohol consumption affects the body as a whole. The immunomodulatory response to the alcohol affects antimicrobial defense and inflammatory responses which results in the prognosis of the disease. Immunometabolism between adipose tissue and systemic metabolism plays a significant role in impairing the insulin uptake [49]. Mechanisms of homeostasis of immune cell-mediated metabolic responses in adipose tissue in ALD are not known. Another study in the rat model suggested mesenteric lymphatic leak, presence of dendritic cells and T_reg_ into perilymphatic adipose tissue, decreased CD4/CD8 ratio in the mesenteric lymph node, and decreased glucose uptake by perilymphatic adipose tissue indicating possible dysregulation of immunometabolism [52].

It is well established that ethanol consumption increases reactive oxygen species (ROS) in a central mechanism, which induces vascular toxicity [53–55]. Acute ethanol consumption is also linked with cardiovascular events [56]. The enzyme NADPH oxidase produces ROS in both vascular and endothelial muscle cells [57]. In the vasculature, acute ethanol intake activates NAD(P)H oxidase which further leads to elevation of O_2_^–^ and lipoperoxidation [55, 58].

Vascular composition such as perivascular adipose tissue (PVAT) is a vital modulator of various agonists in vascular contraction of blood vessels [59, 60]. Thus, PVAT functions as a paracrine modulator for secreting adipocyte-derived relaxing factors (ADRF), which are still not completely characterized [61]. PVAT regulates vasoconstriction through ADRF and can also contract perivascular nerve stimulation as it comprises reactive oxygen species and superoxide anion [62]. In a study of acute ethanol exposure in rats, the observation suggested that PVAT protects against vascular dysfunction through increased production of H_2_O_2_ [56]. This can be a possible new mechanism of deposition of VAT resulting in hypoxia and inflammation within the tissue.

The specific immune response that drives the dysfunction of adipose tissue is not known. Another possible mechanism is the role of toll-like receptor 4 (TLR4) in adipocyte metabolism. Due to deposition of lipids in hepatocytes, the adipose tissue is the second organ being affected in pathogenesis of ALD. Several chronic alcoholic studies reported that PPAR*γ*, CYP2E1, Bid, and C1q are potential mediators for the inflammation in adipose tissue [63–65]. Simultaneously, anti-inflammatory adipokines are decreased in adipose tissue [66]. This phenomenon will trigger the inflammatory state in adipose tissue resulting in metabolic dysfunction of both the adipose tissue and liver [49]. A previous study suggests that in the absence of TLR 4 knocked out, TLR 2 and 9 are involved in the inflammation in adipose tissue [49, 67]. TLR4 expression in a nonmyeloid cell type can switch M1 macrophage phenotype. While in myeloid cells, dendritic cell accumulation is absent when TLR4 is deleted in the presence of alcohol. Accumulation of neutrophil and depletion of CD8^+^ T-cell are not dependent on TLR4. These conclusions help determine a role of adipose tissue inflammation in ALD [68].

## 6. Role of CYP2E1 in Adipose Tissue and Innate Immunity in ALD

Ethanol is metabolized by CYP2E1 leading to oxidative and endoplasmic stress, which alters adipokine regulation leading to prognosis of ALD. 4-Hydroxynonenal, an indicator of oxidative stress, was identified in the adipose tissue upon chronic ethanol feeding [44, 69]. Progression of inflammation in adipose tissue is co-related with the increase of ethanol induced CYP2E1 expression via triggering redox-sensitive transcription factors that leads to increase in ROS production. Moreover, amplified CYP2E1 expression also leads to activation of C1q-dependent complement system and apoptosis facilitated by Bid causing a secondary CYP2E1 facilitated inflammatory response [70]. Simultaneously, levels of macrophage migration inhibitory factor (MIF) and inflammatory cytokines are amplified in alcoholic cirrhosis patients, though the exact mechanism of MIF increase is unknown [71–73]. Disruption of adipokine release from adipose tissue and increase in filtration of macrophages upon intake of alcohol is well known [74, 75]. This results in alteration of adipose tissue accredited to oxidative stress induced by alcohol metabolism [7]. A study indicated that upon chronic alcohol exposure, increased proinflammatory cytokines not only modify the metabolism of adipose tissue but also deregulate adipokine regulation [76].

## 7. Adipokine Regulation upon Alcohol Induction

More than 600 adipokines are secreted from the WAT endocrine organ, which regulates the metabolism of multiple tissues [67]. Among several adipokines, leptin and adiponectin are the major ones which affect the liver.

### 7.1. Adiponectin

It is an adipokine, i.e., anti-inflammatory with insulin sensitizing and adipogenic effects via alteration of AMPK pathway which affects glucose metabolism and fatty acid oxidation in tissues.

The center for ectopic fat storage and lipid storage is through adiponectin. The majority of animal investigations on prolonged alcohol intake show a drop in circulating adiponectin [39, 48, 49, 77–82]. One contradicting study has suggested that the decrease in adiponectin in chronic drinkers (>50 g/day) had no correlation between levels of adiponectin and alcohol intake [83]. Results of adiponectin response in rodents and humans upon alcohol consumption are listed in Table 1. The result discrepancy of/in rodents shows decrease in adiponectin, while in humans' lower doses of alcohol increased adiponectin is species-specific response. A definitive explanation for such a response has not been elucidated.

Potential mechanisms for the decrease of adiponectin in rodents have been identified. In an *in vitro* rat study, VAT cells upon exposure to alcohol MAPK pathway and PPAR*γ* pathway were activated resulting in the decrease of adiponectin secretion [49]. Also, alcohol-treated animals contribute to the impairment of cellular stress and decrease of adiponectin. Alcohol feeding for four days leads to upregulation of CYP2E1 and induction of oxidative stress, including increase of 4-hydroxynonenol (4-HNE) accumulation and a decrease glutathione (GSH/GSSG) ratio [7, 77]. Another investigation presented that intake of 4 weeks of alcohol elevated CHOP mRNA in eWAT and decreased adiponectin [84]. This correlation of ER stress and CHOP may be due to alcohol-induced increase in homocysteine levels, a decline in methylation of *S*-adenosylmethionine (SAM)/*S*-adenosylhomocysteine (SAH) ratio and the enzyme cystathionine *β*-synthase, which is important for the conversion of homocysteine to cysteine in eWAT.

### 7.2. Leptin

Intake of food, energy expenditure, lipolysis, lipogenesis, and fatty acid oxidation are the processes which are regulated by leptin. Since receptors of leptin are present all over the body, it has both paracrine and autocrine functions. Lipid deposition in the liver can be inhibited by activation of *β*-oxidation of fatty acids by leptin hormone [85]. Circulating leptins are correlated with the alteration in fat mass more than the presence of alcohol. There are contradicting reports on chronic alcohol uptake in rodent models; a few suggested increase in leptin [84, 86, 87] while decrease in leptin is also reported [88, 89] and one study observed no change [84]. Therefore, considering the conflicting findings, no clear pattern or consistencies are derived.

In human studies, serum leptin concentration is not correlated with alcohol intake [90–95]; however, there are few studies which demonstrated conversely [96, 97]. The study reported that the fat mass is directly correlated with the serum leptin levels in alcoholics [96].

Increased leptin protein [75] and mRNA [97–99] within adipose tissue of chronic alcohol-fed rats and mice were observed, while in subcutaneous adipose tissue of alcoholic patients, leptin mRNA remains unaffected. Taking together the above findings, it has been concluded that serum leptin level decreases after administration of ethanol under leptin suppression by adipose tissue into the systemic circulation [100].

### 7.3. Resistin

Adiponectin can be suppressed by resistin and stimulate lipolysis to initiate the release of glycerol and fatty acids in blood circulation [101], while in rodent's chronic alcohol increases serum resistin [102]. Similar results were observed in men, the absentia of alcohol for 7 days did not normalize the level of resistin, while alcoholics had little effect in women [103]. In adipose tissue, resistin mRNA expression in rats did not differ, while protein content was increased upon 4 weeks of alcohol induction [102, 104]. Resistin in VAT is increased with 22 weeks of high alcohol feeding (5 g/kg/day), while with lower dose (0.5 and 2.5/g/kg/day) did not alter the resistin level [99]. However, with data being limited, it can be concluded that chronic alcohol is required to raise serum and adipose tissue resistin.

### 7.4. Chemerin and Visfatin

Chemerin is not known for adipokine, but it has important paracrine and autocrine functions in controlling the differentiation and adipogenesis of adipocyte [105]. In studies of the chronic alcohol model of humans and rats, elevation of levels of chemerin is observed in both serum and VAT [106]. In men, levels of chemerin are positively correlated with BMI, body fat levels, and triglycerides [106].

Visfatin is associated with adipose tissue glucose metabolism. A study on rats determines a dose-dependent association with alcohol and expression of visfatin levels in serum and VAT [84]. A regular dose of 5 g/kg/day was required to elevate the plasma visfatin concentrations, while a lower dose of 2.5 g/kg/day did not modify visfatin levels in rat serum [107], though the same constant dose for 3 days did decline the plasma peptide levels [108].

Accumulated evidence supports a chronic alcohol effect on the foremost adipokines, i.e., leptin and adiponectin. Conversely, these effects are not coherent while comparing rodents and humans. The alcohol stimulated modification in adipokine response in various models is presented in Table 1.

**Figure 2:**
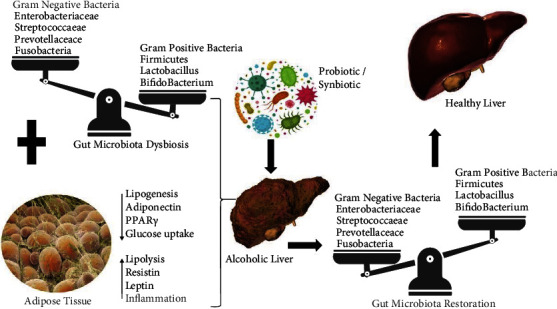
Possible treatment strategy to modify gut-adipose tissue-liver axis by the ingestion of probiotic and symbiotic leading to the restoration of gut microbiota.

A possible treatment strategy to modify gut-adipose tissue-liver axis by the ingestion of probiotic and synbiotic leading to the restoration of gut microbiota is represented in Figure 2.

## 8. Conclusion

Chronic alcohol consumption leads to intestinal dysbiosis in rodents as well as in humans' studies. Altering intestinal barrier function, gut leakiness, triggering proinflammatory cytokines, and pathogenic microbial products lead to endotoxemia which causes liver injury as well as adipose tissue dysfunction. Metabolic changes in adipocytes lead to an impaired lipolysis, glucose metabolism, TLR4 activation, and adipokine secretion leading to the inflammatory environment. These cascades of adipokines are not exclusive to adipose tissue as it affects hepatic steatosis as well as other tissues all over the body. Till date, research implies that prognosis of ALD can be reduced or prevented by improving the function of adipose tissue and gut. Although considerable development has been made in understanding the adipose tissue metabolism in the presence of alcohol, the exact essential molecular mechanisms connecting adipose tissue injury and development of liver disease upon alcohol ingestion need to be elucidated. Adipokines play a vital role in ALD and drug targeting adipokine, and gut pathways needs to be established and tested for prevention and amelioration of ALD. Therapeutic innervations such as probiotics, prebiotics, synbiotics, or polyphenols may alleviate intestinal microbiota composition with better understanding of the intestinal microbiota homeostasis which may be helpful in preventing the prognosis of ALD.

## Figures and Tables

**Table 1:** Adipokine response to consumption of alcohol in rodents and humans.

Model	Effect of alcohol consumption	Reference
Rodent	Decrease in circulating adiponectin	[77]
	Decrease in adiponectin and cartonectin levels	[82, 86, 89, 109–112]
	Increase in leptin, resistin, IL-6, visfatin, chemerin, TNF-a, MCP-1, and RBP-4	[70, 83, 111, 113, 114]
	Decrease leptin	[88, 89]
	Unchanged leptin levels	[99]
	Increase resistin, chemerin, and visfatin	[102, 104, 106]
	Increase in triglyceride gradation and insulin resistance	[115, 116]
Human	Increase plasma adiponectin in chronic alcohol model	[117–119]
	Decrease plasma leptin in plasma chronic alcohol model	[97, 120, 121]
	Increase in leptin, resistin, and chemerin	[99, 103, 106]
	Unchanged leptin and resistin	[90–95, 103]
	Increase in adiponectin, resistin, ghrelin, TNF-alpha, and IL-6	[43, 48, 70, 78, 122–126]
	Decrease in acylation of stimulating protein	[127]
	Glucose intolerance	[97, 128, 129]

## Data Availability

The data relevant to the review article is within the manuscript.
